# Vegetable Oils for Material Applications – Available Biobased Compounds Seeking Their Utilities

**DOI:** 10.1021/acspolymersau.5c00001

**Published:** 2025-03-19

**Authors:** Vojtěch Jašek, Silvestr Figalla

**Affiliations:** † Institute of Materials Chemistry, Faculty of Chemistry, Brno University of Technology, 61200 Brno, Czech Republic

**Keywords:** Vegetable oils, Green chemistry, Triacylglycerides, Material applications, 3D printing, Polyurethanes, Coatings, Adhesives, Biobased materials

## Abstract

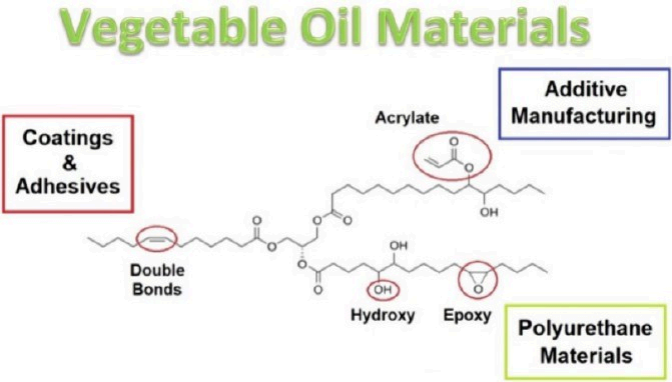

Materials derived from natural sources are demanded for future applications due to the combination of factors such as sustainability increase and legislature requirements. The availability and efficient analysis of vegetable oils (triacylglycerides) open an enormous potential for incorporating these compounds into various products to ensure the ecological footprint decreases and to provide advantageous properties to the eventual products, such as flexibility, toughness, or exceptional hydrophobic character. The double bonds located in many vegetable oils are centers for chemical functionalization, such as epoxidization, hydroxylation, or many nucleophile substitutions using acids or anhydrides. Naturally occurring castor oil comprises a reactive vacant hydroxyl group, which can be modified via numerous chemical approaches. This comprehensive Review provides an overall insight toward multiple materials utilities for functionalized glycerides such as additive manufacturing (3D printing), polyurethane materials (including their chemical recycling), coatings, and adhesives. This work provides a complex list of investigated and studied applications throughout the available literature and describes the chemical principles for each selected application.

## 1 Introduction

**Figure 1:**
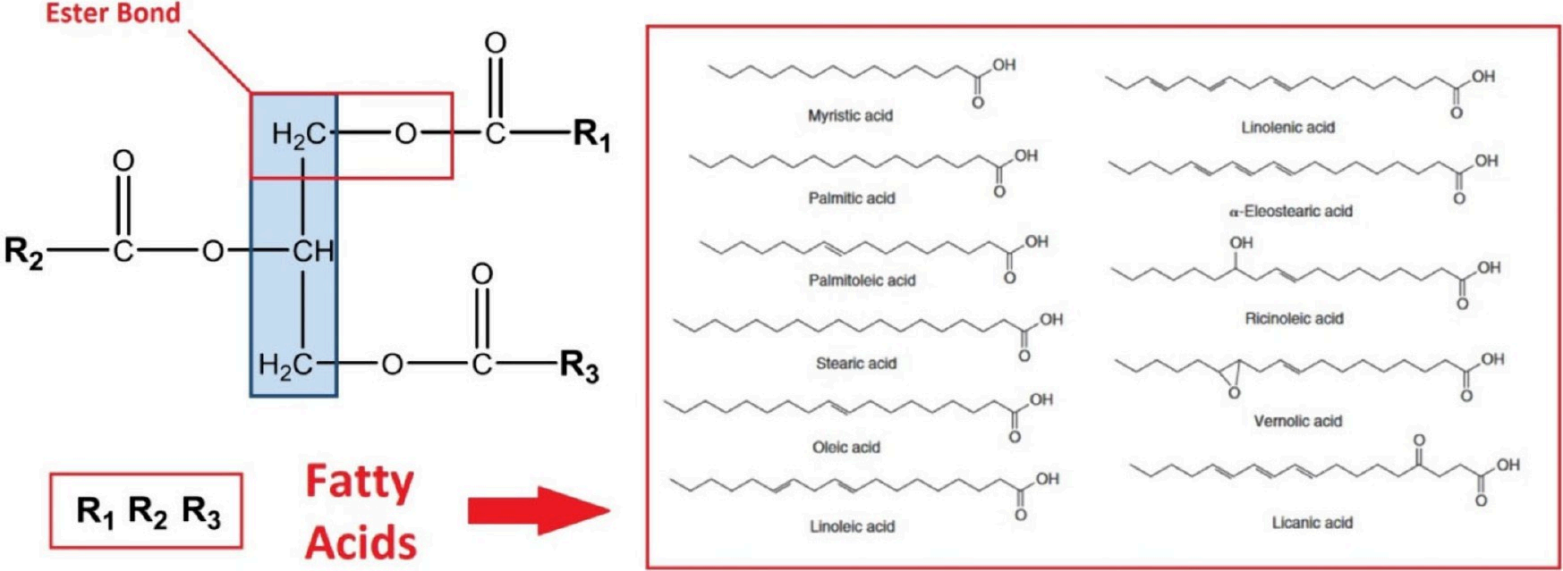
General composition of triacylglycerides involving various occurring fatty acids within their structure. Reprinted or adapted with permission under a Creative Commons (http://creativecommons.org/licenses/by/4.0/) from ref (35). Copyright (2019) Wiley Online Library.

Vegetable oils, fundamentally named triacylglycerides, are complex carbon-containing structures produced by various naturally occurring living organisms across the entire world. Chemically, these molecules comprise a multifunctional alcohol, glycerol, and a wide variety of carboxylic fatty acids. Figure 1 illustrates the different types of fatty acids present in triacylglycerides. Together, an ester structure is formed that possesses physical–chemical properties according to the composition of the particular fatty acids.^1−3^ The majority of vegetable oils share the same characteristics—triacylglycerides contain mostly carbon within their structures (75% or more), and the rest of their mass comprises oxygen and hydrogen.^4^ Triacylglycerides exhibit a significant hydrophobic character ensuring their problematic miscibility with water.^5,6^ Since all vegetable oils are esters, the hydrolysis occurs when optimal conditions are reached. The ester functional group reacts with water in acidic and basic environments forming glycerol in both scenarios and free fatty acids or fatty acid salts depending on the conditions, respectively.^7−9^

Besides the ester functional groups present in all vegetable oils, much particular structure-forming bonding forms the eventual oil’s carbon backbone. The fully saturated carbon alkane structures typically occur in the solid-phase oils such as palm or coconut oil.^10,11^ Solid-phase vegetable triacylglycerides contain mainly myristic (C14), palmitic (C16), or stearic (C18) acids.^12^ Their solid form at room temperature is a consequence of the induced dipole–induced dipole molecular interactions (also known as the dispersion forces or London interactions).^13^ This intermolecular attraction is a fundamental consequence of the inconsistent electron distribution in all of the existing structures. According to quantum-mechanics theory, all molecules (containing or lacking permanent dipoles) contain electrons, whose trajectory and position are described by the Schrödinger equation. The dispersion interactions are generated due to the inconsistent electron position within the chemical structure, causing attractions between molecules. The dispersion forces exhibit short-range attraction; therefore, a minimal distance between compounds is mandatory to maximize the London forces. The most discussed and agreed distance for dispersion interactions is 1/*r*^6^, where *r* (m) stands for the radius of two compounds exhibiting the interaction.^14−16^ The fully saturated fatty acids contained in solid-phase oils at room temperature can reach the mandatory attraction distance due to the nonlimited molecular rotation of single bonded alkanes. Therefore, the maximum dispersion forces effect may be manifested.^17^ On the other hand, oils possessing unsaturated double bonds within their structure (composed of sigma and pi bonds) appear as liquid systems at moderate temperatures.^18^ Liquid state vegetable oils primarily contain unsaturated carboxylic acids such as oleic (C18, one double bond), linoleic (C18, two double bonds), or linolenic (C18, three double bonds) acid.^19−21^ These systems are liquid at the same temperature as the solid oils containing saturated fatty acids due to the free molecular movement limitation caused by the occurring double bonds. The unsaturated carbon backbone segments do not exhibit free molecular movement and rotation; therefore, the carbon orientation is not isotropic. The anisotropic character of the unsaturated acids in liquid oils causes a lesser opportunity to form dispersion forces between compounds structures. The double bond determines the fixed form of the carboxylic acid. Therefore, the short-range London forces cannot be manifested in the same quantity as in the case of saturated fatty acid oils. The lesser dispersion force attraction leads to the remaining liquid state form of such systems. The more attractive molecular interactions formed, the higher the critical temperatures such as melting or boiling points.^22,23^

The saturated and unsaturated fatty acids play a major role in the determination of the eventual material properties. However, there are specific vegetable oils containing unique carboxylic acids within their structure possessing particular additional functional groups, which may be used for suitable applications. Castor oil has a specific fatty acid composition, including mostly ricinoleic acid. This compound possesses a C18-long backbone with one double bond (similar to the oleic acid).^24,25^ However, a vacant hydroxyl functional group occurs in the carbon chain, which promises a unique utility potential. Reactive hydroxyl groups plays a major role for certain material matrices (polyethers, polyesters, or polyurethanes)^26−28^ and can be modified by an appropriate substance (e.g., esterified by an acid).^29^ Also, this structural composition increases the polar character of this particular triacylglyceride, which can result in specific semipolar requiring applications.^30^ Vernolic acid is another specific compound which can be found in biobased sources. This fatty acid contains a C18-long carbon chain, one unsaturated double bond, and one epoxy functional group.^31^ This reactive species is uniquely found in biological systems. There are particular plants comprising this fatty acid such as *Vernonia galamensis*, *Euphorbia lagascae*, and *Crepis palaestina*.^32,33^ The epoxy group naturally occurring in this triacylglyceride can be a promising reactive center for nucleophilic substitutions or a potential cationic polymerization.^34^

This presented Review summarizes specific materials and polymeric products applying biobased complex systems (vegetable oils, itaconic acid, itaconic anhydride, hydrolyzed glycerides) to their technologies, processes, and manufacturing. The increase of renewable content in the production is the main linking parameter for all described utilities, since the legislature, namely in the European Union, tends to set new standards for future products and distributed products which will need to incorporate biobased or recycled content.^36^ Also, vegetable oils are produced, harvested, and available across the globe; therefore, their role in manufacturing is strongly beneficial and can be implemented in various geographic regions. Soybean, palm, and rapeseed oils are primarily produced and used in various applications due to their high yields and sufficient stability. Recent sources report that soybean oil represents approximately 60% of total oil production.^302^ Triacylglycerides were produced in 212.82 million tons in 2022.^302^ Additionally, several secondary products possess a triacylglyceride structure such as used hydrolyzed and oxidized waste cooking oil,^37^ mono and diglycerides of waste cosmetic oil,^38^ or various extracts and byproducts from ethanol manufacture or pure coffee ground production.^39,40^ The biobased byproducts and waste manufacture incorporation leads to more rentable and sustainable industry and fulfills future legislature requirements.

## 2 Additive Manufacturing

The reactive functional groups such as a vacant hydroxyl in the castor oil’s structure, available hydroxyl groups present in unbonded hydrolyzed glycerol, or modified double bonds in unsaturated triacylglycerides play major role for potential vegetable oils’ utility in additive manufacturing (3D printing).^41,42^ The oil structure’s availability can be natural (castor oil), purposefully modified (using double bonds as reactive centers), or used in the waste hydrolyzed oil (vacant hydroxyls from the unbonded glycerol).^41,42^ Typically, vegetable oils have enormous potential for stereolithography (a liquid curable system is used as a resin precursor in 3D printing), since these systems are liquid at moderate temperatures.^43^ Powder bed fusion 3D printing is usually not optimal for oil-involving systems. The particular systems based on different chemical working principles are listed, introduced, and discussed below to summarize a complex viewpoint on the modified curable oil’s potential for this utility.

### 2.1 Epoxidized Oils

**Figure 2:**
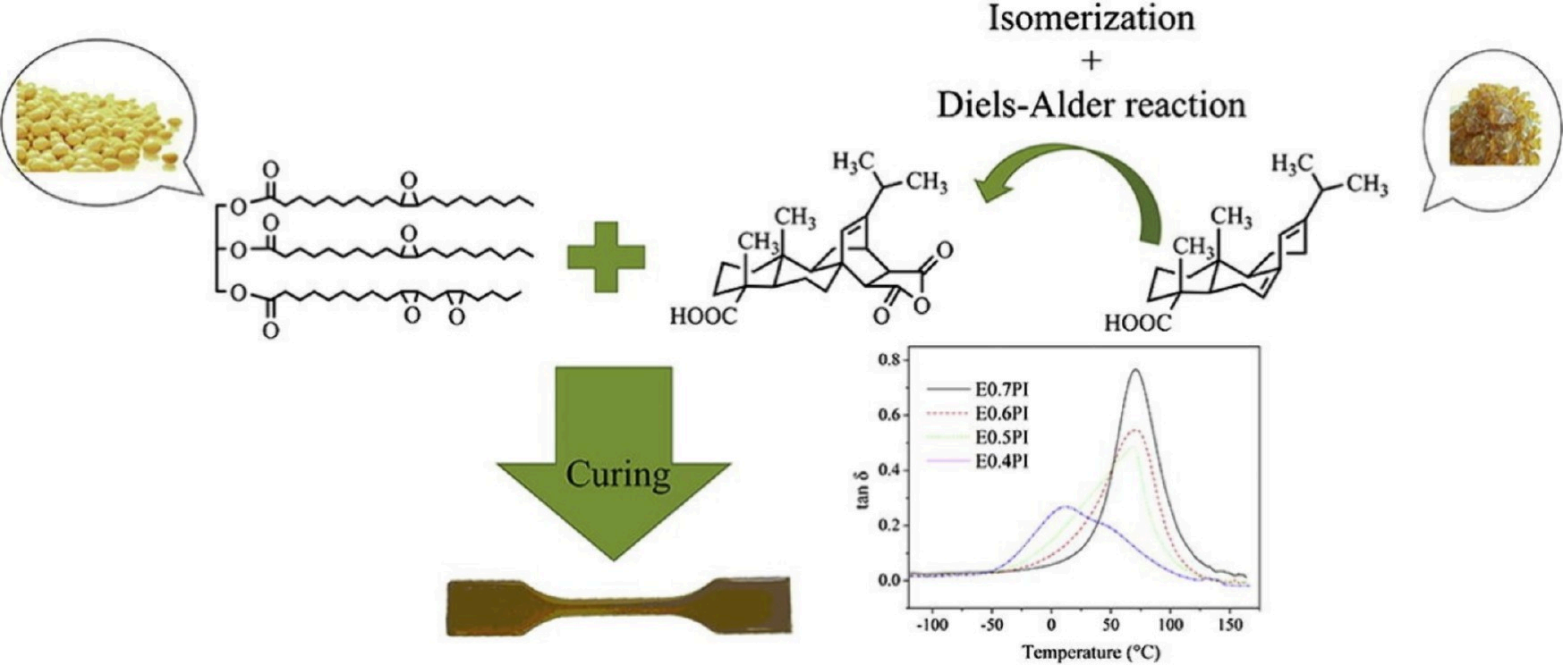
Direct application of epoxidized vegetable oil for curable thermoset production through ring-opening polymerization of epoxy functional groups in soybean epoxidized oil. Reproduced with permission from ref (54). Copyright (2016), Elsevier.

Two-step modification of double bonds within vegetable oil’s structure using epoxidation leading to the acrylated and methacrylated triacylglycerides has been reported numerous times in the literature.^44−48^ The particular epoxidized vegetable oil’s chemical structure leading to the cured thermosets is displayed in Figure 2. Unsaturated bonding can be oxidized purposefully (obtaining epoxy functional groups serving further perspective modification),^49^ while spontaneous oxygen modifies these bonds to peroxides leading to the oil’s structural degradation.^50^ The epoxy groups’ incorporation to a triacylglyceride backbone was succeeded via numerous reactive systems (see Table 1). The most well-known and studied epoxidation approaches use 3-chloroprebenzoic acid (mMCPA). This reactant works with various different entering compounds comprising double bonds within their structure. However, mCPBA reacts in an equimolar manner to the number of double bonds, which leads to its high mass consumption. Also, 3-chlorobenzoic acid is formed during epoxidation, which complicates the production since additional purification steps ensuring the disposal of this compound must be performed.^51−53^

**Table 1:** Oil Epoxidation Approaches

Type of vegetable oil	Epoxidation mixture	Molar ratio C=C to epox. agent	Molar ratio C=C to H_2_O_2_	Catalyst	Reaction temperature (°C)	Reaction time (h)	Reference
Soybean oil	H_2_O_2_ + CH_3_COOH	1:0.5	1:1.5	H_2_SO_4_	65	4	(58)
Cotton seed oil	H_2_O_2_ + CH_3_COOH	0.25:0.75	1.1:2.5	Strong Inorganic Acid	60	4	(59)
Castor oil	H_2_O_2_ + CH_3_COOH	1:5.5 (wt)	1:1.61 (wt)	Seralite SRC-120	55–60	8	(65)
Grape seed oil	H_2_O_2_ + CH_3_COOH	1:0.5	1:2	H_2_SO_4_	60	12	(60)
Sesame seed oil	H_2_O_2_ + HCOOH	1:0.8	1:3.5	H_2_SO_4_	80	6	(63)
Rapeseed oil	H_2_O_2_ + HCOOH	1:0.75	1:3	H_2_SO_4_	70	3.3	(62)
Palm kernel oil	H_2_O_2_ + HCOOH	1:0.85	1:1.46	/	40	2	(64)
Camelina sativa oil	H_2_O_2_ + HCOOH	1:0.66–1.2	1:0.85–1.7	/	50	5	(61)
Free fatty acids	mCPBA	2:1 (wt)	/	/	RT	0.16	(69)
Glyceryl trioleate	mCPBA	1:1 (wt)	/	/	RT	1	(67)
Waste cooking oil	H_2_O_2_ + CH_3_COOH	1:0.5	1:2	Amberlyst 15	60	6	(66)
Waste cooking oil	mCPBA	1:1.1	/	/	RT	90	(66)

The application of other percarboxylic acids is a more prospective, available, and efficient than approach with mCPBA. Many different epoxidation systems were suggested and investigated in the literature,^58−66^ leading to modified vegetable oils. The most promising ones involve performic and peracetic acids. These compounds are available in industrial quantities promising the up-scaling potential.^58,62^ Since both structures exhibit much lesser molecular weight compared to mCPBA (performic acid molecular weight is 62.06 g/mol and peracetic acid molecular weight is 76.05 g/mol), the producing mass quantities and reaction ratios also favor them compared to 3-chloroperbenzoic acid (molecular weight of 172.57 g/mol).^55^ The generation of percarboxylic acids is usually continual, so these compounds immediately react with double bonds.^55^ This approach is commonly employed in aqueous hydrogen peroxide solutions. Epoxidation mixtures usually comprise aqueous hydrogen peroxide in molar excess to the number of double bonds (to ensure a faster rate of the reaction and, in the case of hydrogen peroxide, decomposition), particularly carboxylic acid, and additional stronger acid to provide an efficient percarboxylic acid formation.^58−66^ The assistance of an extra acid is required especially when peracetic acid is included in an epoxidation mixture.^59,60^ Reportedly, performic acid can be generated only in a H_2_O_2_ aqueous solution. Therefore, performic acid formed from formic acid in aqueous solution seems to be to most optimal epoxidation system for oil modifications.^61,64^ Since vegetable oils exhibit strict hydrophobic character due to the absence of polar functional groups, the epoxidation proceeds in a heterogeneous emulsion of water in oil type, where the chemical modification of the double bonds occurs on the phase interface.^56^ This phenomenon ensures oil’s epoxidation efficiency compared to other unsaturated reactants.^56,57^

### 2.2 Hydroxylated Oils

**Figure 3:**
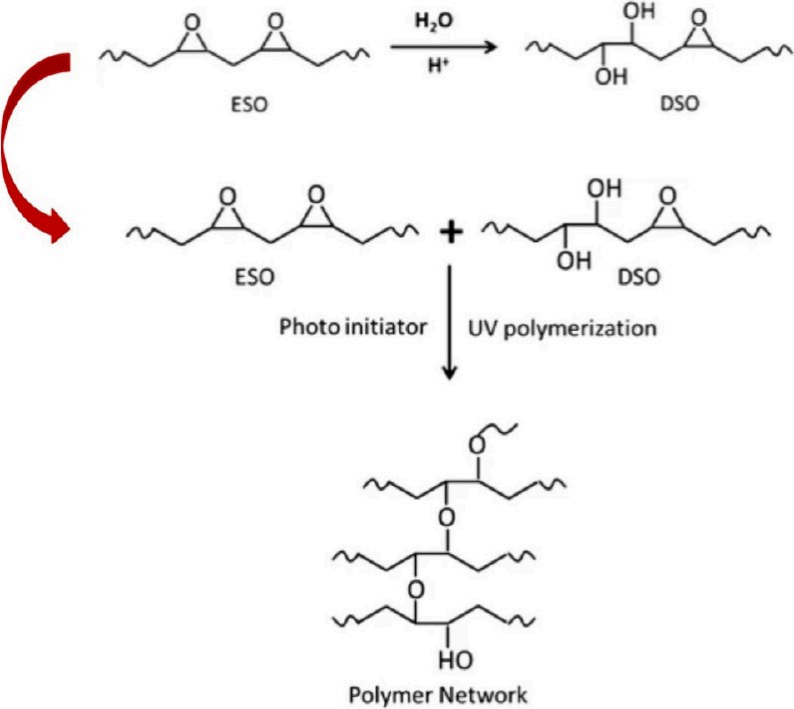
Photoinitially polymerized hydroxylated oil structure. ESO – epoxidized soybean oil, DSO – Dihydroxylated soybean oil. Reproduced with permission from ref (77). Copyright (2014), Wiley.

The epoxy functional groups in the triacylglyceride structure represent reactive centers for nucleophilic substitution. Various nucleophiles for different eventual functional groups are incorporated in oil’s structure.^68^ The full hydroxylation can be reached when water stands for the attacking nucleophile.^70^ A similar reaction forming partially hydroxyl groups and also alkoxy groups uses aliphatic alcohols as nucleophiles.^71^ The hydroxylation increase triacylglyceride’s polar character, since numerous hydroxyls containing a vacant unbonded electron pair increase the permanent dipole of such modified oils.^72^ Next to the electron density rise ensuring the changes in physical–chemical properties connected to solubility, hydrophilic character, or viscosity, the comprised hydroxyl groups may be further chemically modified via esterification.^73^ Numerous carboxyl functional groups can be included in the hydroxylated oil’s structure using different functional derivatives such as alkylhalides,^73^ anhydrides,^74^ or pure carboxylic acid.^75^ This approach may produce a highly modified oil structure containing many neighboring curable acyls useful for an exceptionally cross-linked resin. Such reactive compounds may serve as a cross-linkers and hardeners for other polymerizable systems.^76^ The UV-initiated polymerization providing highly cross-linked structures is schematically illustrated in Figure 3.

### 2.3 (Meth)acrylated Oils

The most discussed, investigated, and experimentally verified approach uses directly acrylic or methacrylic acid as a single-functional carboxyl reacting via nucleophile substitution with an epoxy functional group in modified vegetable oils.^78−80^ This process has several advantages over the direct esterification of available hydroxyl groups involved in native castor oil or hydroxylated triacylglyceride described in the previous section. The rapid epoxy group reactivity promotes the nucleophile substitution at different conditions, usually connected to lower reaction temperatures and duration while yielding the same or higher product yield.^81^ Particular acrylated and methacrylated vegetable oil syntheses are summarized and described in Table 2. The increased rate of nucleophile substitution involving epoxy group and carboxylic acid is also a consequence of removing water formation during the process compared to the standard Fischer esterification.^82^ When acrylic or methacrylic acid reacts directly with the epoxy functional group, the nucleophile is bonded to the triacylglyceride backbone and, simultaneously, the hydroxyl functional group is generated eventually. Therefore, no solitary secondary products are formed during the process.^83^ On the other hand, while a vacant hydroxyl group is esterified using carboxylic acid as a nucleophile donor, water is formed as a secondary product. According to Le Chatelier’s principle, a separation method to remove water continually must be included to ensure a sufficient reaction rate and acceptable conversion. Otherwise, the reaction equilibrium is reached at low product yield, and the process is inefficient.^84^ The water removal is provided via various approaches, which were experimentally verified in numerous reactions. The most used way includes the Dean–Stark apparatus in the synthesis procedure. The principle comprises azeotropic distillation continually removing water in a vapor azeotrope using an appropriate cosolvent. The distilled vapors condense in a Dean–Stark apparatus, water is separated at interphase interface, and the pure cosolvent is returning into the reaction batch.^85^ Although this process is widely used in laboratory-scale experiments,^86−88^ it is not optimal for up-scaled processes, since the energetic requirements are excessive, and major VOC’s quantities may be detected.^89^ Another water-removal approach involves molecular sieves suitable for the encapsulation of water molecules. Such systems are usually manufactured from potassium–sodium aluminosilicates (zeolites), which have a defined pore size. For the water separation, 2.8–3 Å pores are optimal. Once the water molecules are incorporated into the zeolite structure, the molecular sieve must be regenerated usually at high temperatures.^90^

**Table 2:** Rheological and Thermomechanical Properties of Curable Vegetable Oils

Modified oil	Apparent viscosity (mPa·s)	Storage modulus (MPa)	Glass transition temperature (°C)	Catalyst	Reaction temperature (°C)	Reaction time (h)	Reference
Acrylated canola oil	/	435.4 (25 °C)	46.4	Boron trifluoride ether	80	2	(111)
Acrylated soybean oil	4800 (30 °C)	303 (30 °C)	<30	/	/	/	(113)
Acrylated grapeseed oil	3152 (25 °C)	2.2 (30 °C)	–1.7	Boron trifluoride ether	80	5	(115)
Acrylated epoxidized soybean oil	29,100 (25 °C)	<100 (25 °C)	14	/	/	/	(112)
Methacrylated dimeric acids	3500 (25 °C)	287.5 (25 °C)	22.3	4-Dimethylaminopyridine (DMAP) with glycidyl methacrylate	90	5	(114)
Methacrylated epoxidized castor oil	600 (20 °C)	700 (25 °C, + 16 wt % of methacrylic acid)	60 (+ 16 wt % of methacrylic acid)	Triethylamine (TEA) (methacryloyl chloride)	0	0.5	(116)

**Figure 4:**
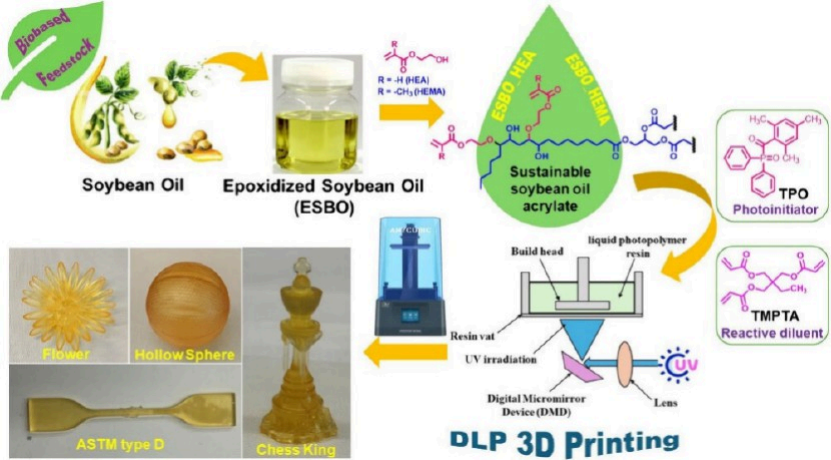
Acrylated soybean oil was used for additive manufacturing. Reprinted with permission from ref (100). Copyright (2024), American Chemical Society.

The acrylated and methacrylated vegetable oils possess signature physical–chemical properties connected directly to their molecular backbone structures. All such modified oils exhibit high viscosity levels caused by the combination of different factors.^91,92^ Modified triacylglycerides have relatively high molecular weight (1000–2000 g/mol) causing an excessive promotion of dispersion forces.^93^ Unmodified vegetable oils containing double bonding usually exhibit a moderate rheological profile (apparent viscosity reaching values in hundreds mPa·s) due to the limited London interaction manifestation.^94^ When the vegetable oil’s unsaturated bonding is modified by the discussed nucleophile substitution, the molecular mobility is changed and may contribute to the eventual system’s viscosity increase.^95^ The most enormous rheology-affecting contribution lies in the vacant hydroxyl functional group formation during the nucleophile substitution involving acrylic/methacrylic acid and the reactive epoxy group. The −OH group is a center of strong electron density caused by the vacant electron pair in the oxygen atomic structure.^96^ Therefore, this structural modification in the triacylglyceride’s backbone leads to the increased dipole–dipole force manifestation (also known as Keesom forces). Whenever more intermolecular forces are generated, the physical–chemical profile changes including the melting of boiling points, and most importantly, the viscosity is also affected.^97^ Furthermore, unbonded hydroxyl groups can form hydrogen bonding when the appropriate proton acceptor is available in the targeting structure. Considering the hydrogen bonding forces, the proton (hydrogen) donor is the hydroxyl functional group containing the covalently bonded hydrogen atom. The hydrogen bonding acceptor is an electronegative atom or functional group typically containing vacant electron pairs, which promotes the hydrogen bonding. Since acrylated and methacrylated oils contain many esterified oxygen atoms, the hydrogen bonding initiated by the formed hydroxyl groups generated after the nucleophile substitution can significantly contribute to the eventual rheology profile transformation.^98,99^ DPL 3D printed prototypes based on acrylated soybean oil are illustrated in Figure 4.

Next to the increasing viscosity with the reactive functional group occurrence, the particular thermal and thermomechanical properties are also specific for the cured oil-based resins used for 3D printing. Since acrylate or methacrylate derivatives are occurring numerously in the oil’s backbone structure, the cross-linking density is usually very high, and the formed cured resin exhibits exceptional toughness and low molecular shrinkage.^101,102^ This phenomenon can be further increased when the vacant generated hydroxyls are additionally modified by methacrylic anhydride (for example, see ref (103)). The curable groups’ occurrence increases the contribution to the overall thermomechanical profile of the eventual 3D-printed resin, increasing its storage modulus, glass transition temperature, and complete toughness.^104−106^ Additionally, the complex highly cross-linked and cured triacylglyceride structure possesses exceptional thermal stability and heat-resistant character. This property was numerously experimentally verified and discussed in many published studies available in the literature.^107−109^ Since stereolithography 3D printing does not require high thermal stability due to the processability at moderate temperature, this property might be more often used in the SLA-printed and carbonized materials for further particular applications such as for heterogeneous catalyst carriers.^110^ Detailed and defined various porous structures with the treated and modified surfaces serving as reaction moderators can be initially fabricated by additive manufacturing. Then, these systems undergo carbonization, which ensures the optimal future carrier structural composition and appropriate surface profile. Eventually, such systems turn into the heterogeneous catalyst. The thermal properties of these resin-forming precursors are one of the key parameters.^110^

### 2.4 Oils Containing Itaconic Acid

Itaconic acid is a special candidate for curable vegetable oil syntheses. This dicarboxylic acid contains a reactive double bond within its backbone structure.^117^ Unlike the other unsaturated carboxylic (crotonic acid) and dicarboxylic acids (maleic or fumaric acid), this compound exhibits reactivity toward radically initiated polymerization.^118^ This property is similar to an acrylic or methacrylic system, while itaconic acid can be obtained through sustainable production.^119^ Therefore, the curable systems based on this compound or its derivatives can succeed with quantitative biobased character. Itaconic acid occurs as a solid substance at moderate temperatures unlike the liquid acrylic or methacrylic acid.^119^ This property complicates its potential for stereolithography, since liquid curable systems are mandatory for this application. However, the appropriate derivatives or used systems for the resin precursors’ fabrication can overcome this complication.^120^

**Figure 5:**
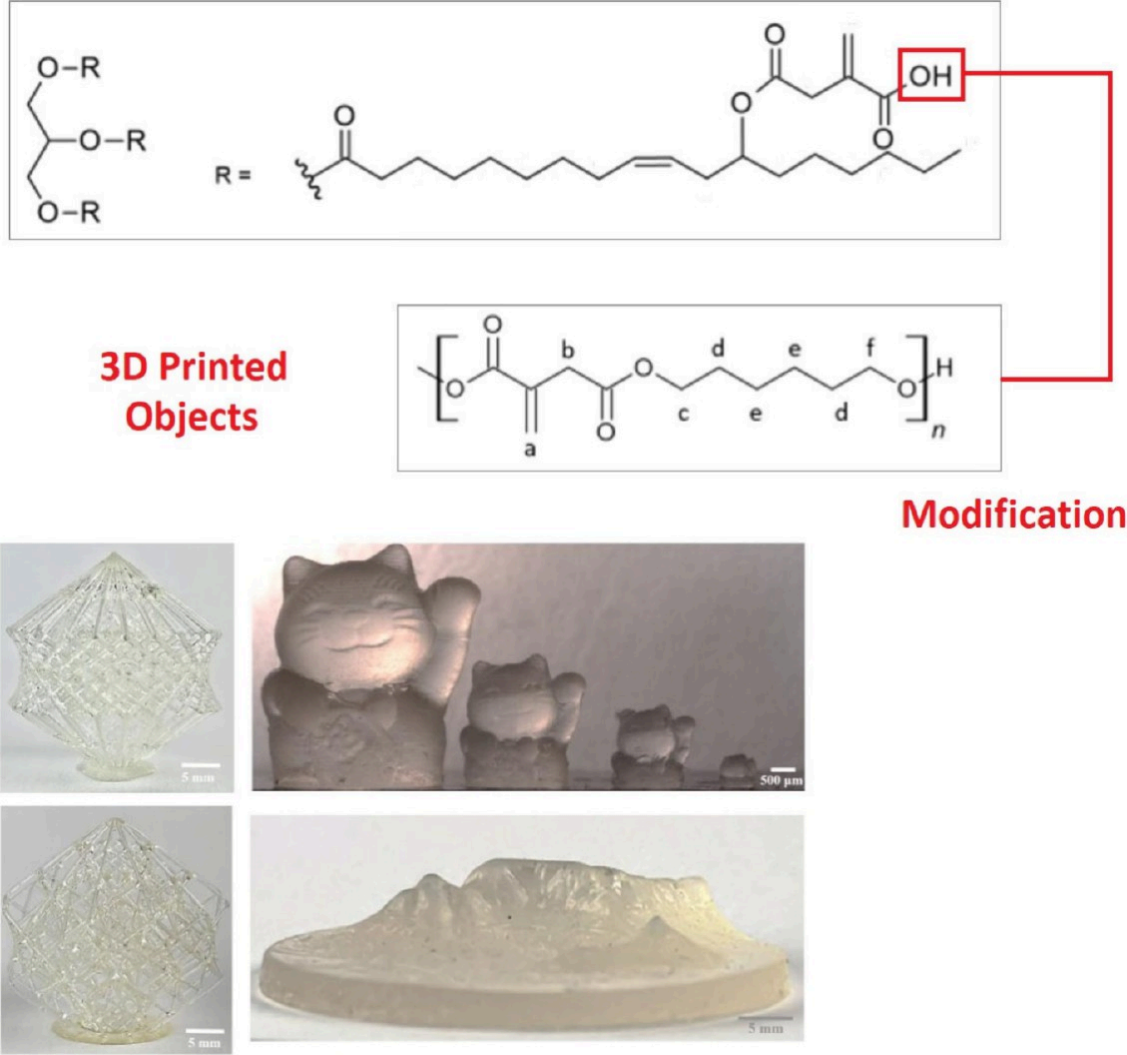
Itaconic acid-modified vegetable oil for 3D printing.^121^ Reprinted with permission under a Creative Commons (http://creativecommons.org/licenses/by/4.0/) from ref (121). Copyright (2024), MATEC Web of Conferences.

The direct esterification of vacant hydroxyl functional groups within triacylglyceride’s structure can be performed using itaconic acid as a nucleophile similarly to the acrylic and methacrylic derivatives production. This process is connected with the byproduct separation, as was discussed earlier. Itaconic anhydride is used more often as a nucleophile for these reactions. Dicks et al.^121^ performed the oil functionalization with itaconic anhydride. Since this molecule appears in a cyclic monomolecular form (unlike the methacrylic or acetic anhydride composed of two separate carboxylic acid molecules, for example), Le Chatelier’s principle cannot complicate the synthesis process. The anhydride molecule forms the covalent bonding with the hydroxyl group, and no byproduct is generated.^122^ However, this process involves the generation of free acidic groups as the cyclic anhydride structure opens (illustrated in Figure 5), which may result in unwanted eventual character of the produced 3D printed product. The vacant reactive polar functional groups, such as carboxylates, promote the potential deprotonation, leading to the pH changes during the contact with particular environments.^121^ Additionally, the hydrophilic character of such precursors and cured resins increases enormously, which is usually negative for an additive manufacture product. The fabricated objects absorb water vapor, leading to swelling, which is an adverse property in this application. The reactive vacant carboxylic groups can be eliminated by additional functionalization with various alcohols of anhydrides leading to the higher hydrophobicity and better applicability.^123^

Another approach using itaconic acids for curable oil systems consists of mixing the synthesized systems with this dicarboxylic acid, such as polyesters, with functionalized vegetable oils.^124,125^ These multicomponent precursors can from the cured thermoset incorporate both structures into the complex molecular site. Since itaconic acid possesses two reactive carboxylic groups, the single-group functionalization may require multistep procedures. When the separate polyester biobased structure is suggested, synthesized, and mixed with the particular oil-based system, the eventual produced thermoset can be obtained more efficiently while having even better material properties than simple modified vegetable oil.^125^ The available polyols such as ethylene glycol, butanediol, or hexanediol can be turned into the oligomer polyester structures with itaconic acid, to ensure the curability, and with other biobased dicarboxylic acids, such as furandicarboxylic or fumaric acid, to increase the complexity of the structure and particular material properties.^126,127^ Compared to the simply modified vegetable oil directly by itaconic acid, these multicomponent reactive mixtures can reach less hydrophilic character, better thermomechanical properties, and overall higher applicable potential.^128^

### 2.5 Oils Modified by Anhydrides

**Figure 6:**
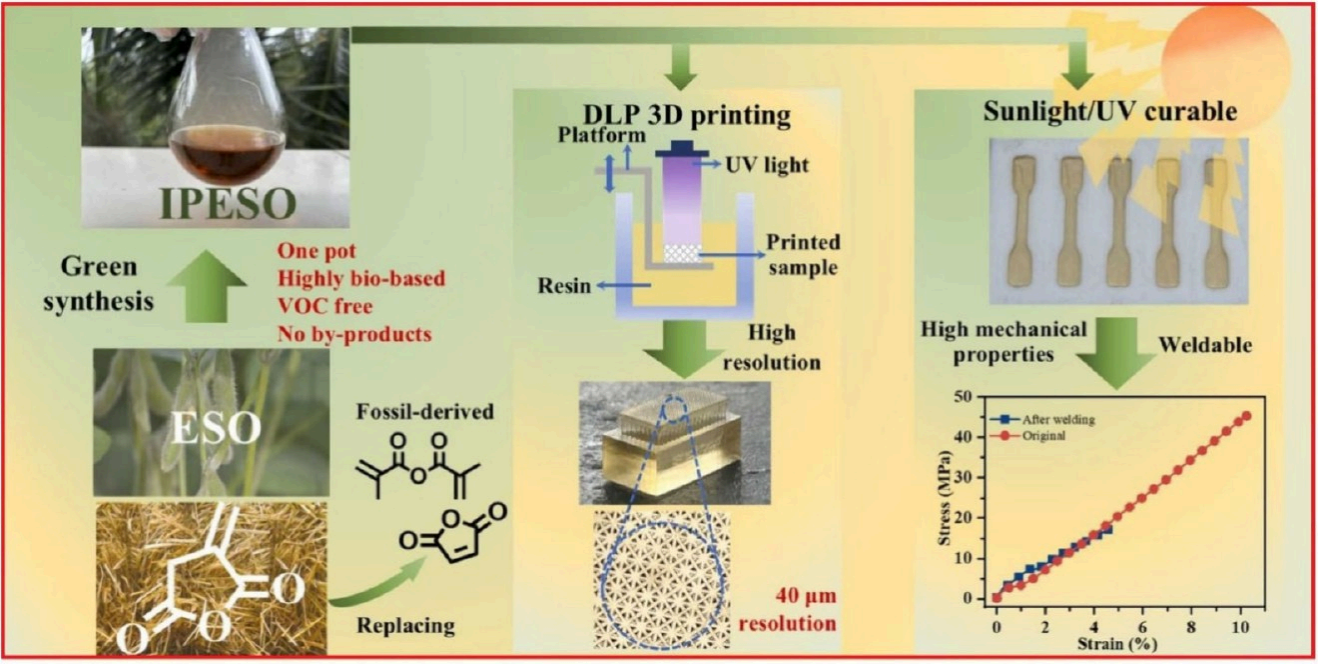
Cyclic anhydride alternative to methacrylic anhydride functionalization of epoxidized soybean oil for DLP 3D printing. Reproduced with permission from ref (134). Copyright (2024), Elsevier.

Most anhydride-containing vegetable oils use one of the two activation approaches of the unsaturated vegetable oil structure’s modification: the epoxidation of double bonds^129^ or the hydroxylated oil’s backbone.^130,131^ The direct esterification or anhydride-involving nucleophile substitution of functionalized triacylglycerides can be successful with itaconic acid^130^ and anhydride,^121^ respectively. The general reaction approach can use different types of reactive anhydride nucleophiles such as maleic or gutaric anhydrides.^132,133^ The anhydride-incorporation approach is illustrated in Figure 6. Such incorporated compounds react with epoxy or hydroxyl functional groups, resulting in the esterified or anhydride-modified triacylglyceride structure capable of the free-radical polymerization. The adverse effects of such synthesis strategies were discussed: the presence of a free carboxyl after the functionalization may increase the hydrophilic character, rendering the swelling and further undesired reactivity of acidic carboxylic functional groups as inevitable.^121^

The second anhydride-using approach works with the maleic anhydride attachment on the vegetable oil’s carbon backbone structure via addition.^135,136^ This reaction approach works at much higher temperatures compared to the discussed acrylation, esterification, or nucleophile substitution through epoxy groups.^135,136^ Yu et al.^137^ synthesized the maleated vegetable oil for curable applications. The resulting modified triacylglyceride structure comprises unopened maleic anhydride structures added to the fatty acid chain. Such functionalized molecules do not exhibit much hydrophilic character or exceptionally high viscosity, since the unsaturated bonding remains in the oil’s chemical structure. The swelling is limited mainly due to the absence of any free hydroxyl or carboxyl functional groups—the anhydride remains cyclic after the synthesis, and the hydroxyl functional group is not formed due to the fundamental of the addition reaction.^138^

## 3 Polyurethane Materials

Polyurethanes are produced via the polyaddition of various polyols with isocyanates. In general, many different structures containing hydroxyl groups are used in the same mixture to ensure the optimal properties of the eventual product.^139,140^ Therefore, various long chain diols (polyethers, polyesters)^141,142^ or several cross-linkers (glycerol, pentaerythritol)^143,144^ compose the polyol mixture used for polyurethane synthesis. Isocyanates also vary, depending on the particular application. Toluene diisocyanate (TDI) or methylene diphenyl diisocyanate (MDI)^145^ and their oligomers, prepolymers,^146,147^ and derivatives are widely used to produce polyurethanes. The application field of this polymeric structure is broad. Thermoset polyurethane is used mainly as foam-forming material for several thermal insulators,^148^ noise-preventing materials,^149^ or interior components.^150^ Particularly, functionalized polyurethane-forming components also serve as adhesives or glues.^151,152^ Thermoplastic polyurethane (TPU) stands for specific application field differing from thermosetting systems. Linear-chain polyurethane composes several car interior components,^153^ operation panels and gadgets,^154,155^ and gear knobs.^156^ Considering the triacylglyceride involvement in polyurethane materials, any vacant hydroxyl functional groups occurring in different oil structures hold an essential potential in turning these commonly petroleum-based systems into more sustainable and biobased substances.^157^ Many natural and functionalized oils can serve as direct polyols for the polyaddition or play an important role in polyurethane chemical recycling.^158^ Several application possibilities are discussed in this section.

### 3.1 Castor Oil

**Figure 7:**
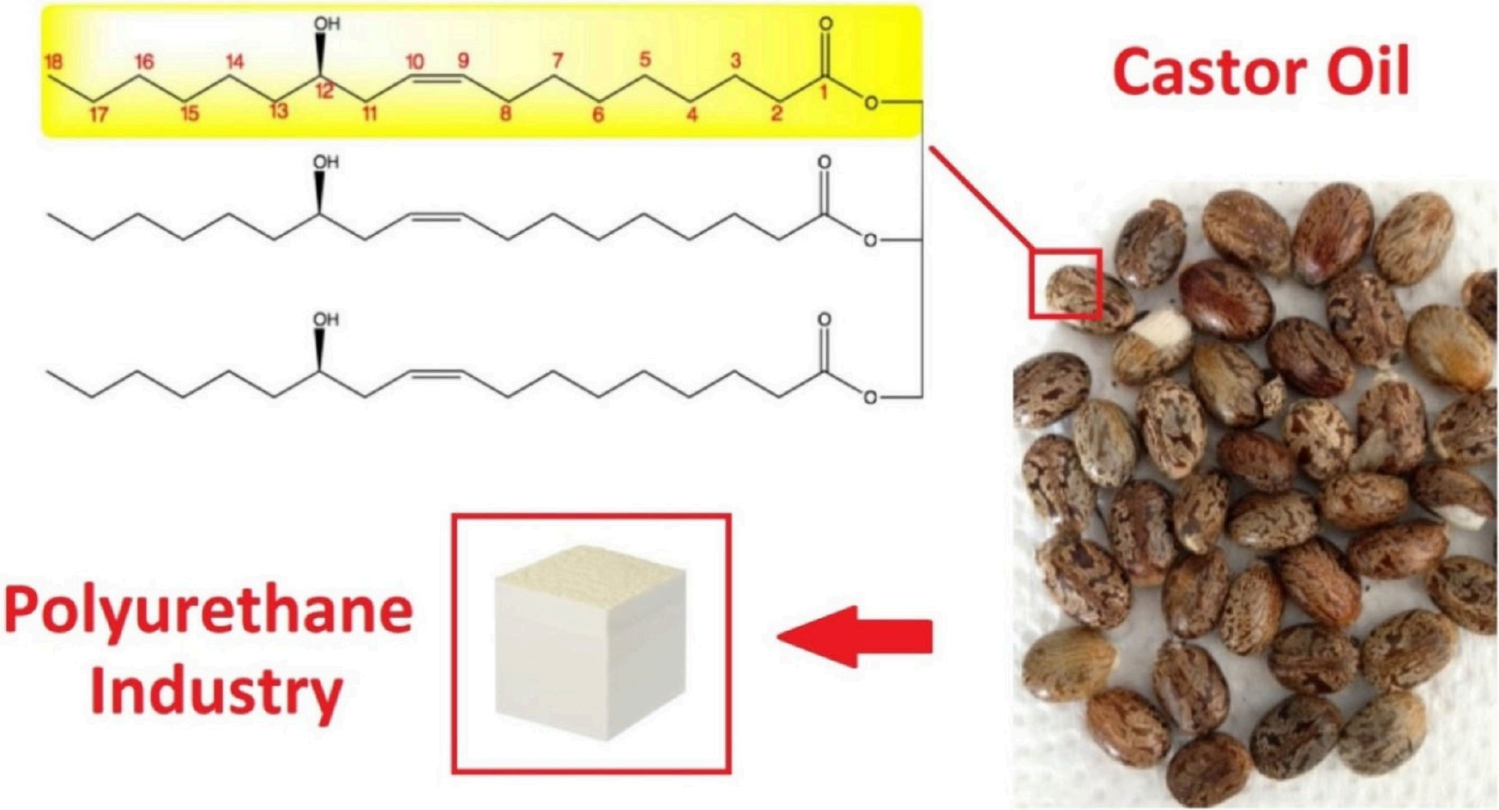
Advantageous castor oil structure for polyurethane manufacturing. Reprinted with permission under a Creative Commons (https://creativecommons.org/licenses/by-nc/3.0/) from ref (159). Copyright (2016), SAGE Publications.

Castor oil, involving ricinoleic acid, has been discussed frequently in this Review. The addition of this triacylglyceride to polyurethane materials has been widely investigated.^168−174^ Next to the most well-known foam-forming materials, castor oil-based products serve various other applications such as fuels, biodiesels, soaps, waxes, lubricants, coatings, or fertilizers.^160−166^Table 3 summarizes various application fields for castor-oil-incorporated PUR materials. The unique structure suitability of this particular triacylglyceride lies in the −OH group located on the C12 carbon, as displayed in Figure 7 on the simplified castor oil’s triacylglyceride structure (castor oil contains minor nonricinoleic acid content). The atomic distance between each hydroxyl is long enough to expect flexibility character enhancement and chain-extending effects. Additionally, the presence of one double bond within ricinoleic structure ensures the liquid appearance at moderate temperatures as was explained earlier in this Review.^167^

**Table 3:** Castor Oil in Polyurethane Applications

Castor oil in polyurethane industry						
Application	Castor oil form	Weight castor oil content (%)	Function	Oil source	Verified scale	Reference
Interpenetrating polymer networks (IPN)	Transesterified oil	90–10	Increase of glass transition temperature	Market	100–1000 g	(168)
Flexible foam	Glycerolyzed	50	Flexibility enhancement	Market	10–30 g	(169)
Coatings	Ricinoleic acid derivative	50	Sustainability increase	Market	100 g	(171)
Adhesives	Triacylglaceride	5–7	Cross-linker substitute	AR, Macklin Reagent Co.	Laboratory	(170)
Nonisocyanate polyurethane	Triacylglaceride	33–25	Sustainability increase	Market	Laboratory	(174)
Thermoset elastomeric films	Functionalized triacylglyceride	30–40	Electrode material	Sigma-Aldrich	Laboratory	(172)
Thermoplastic polyurethane (TPU)	Aminolyzed triacylglyceride	20 (mol.%)	Sustainability increase	Market	Laboratory	(173)

### 3.2 Hydrolyzed Glycerides

**Figure 8:**
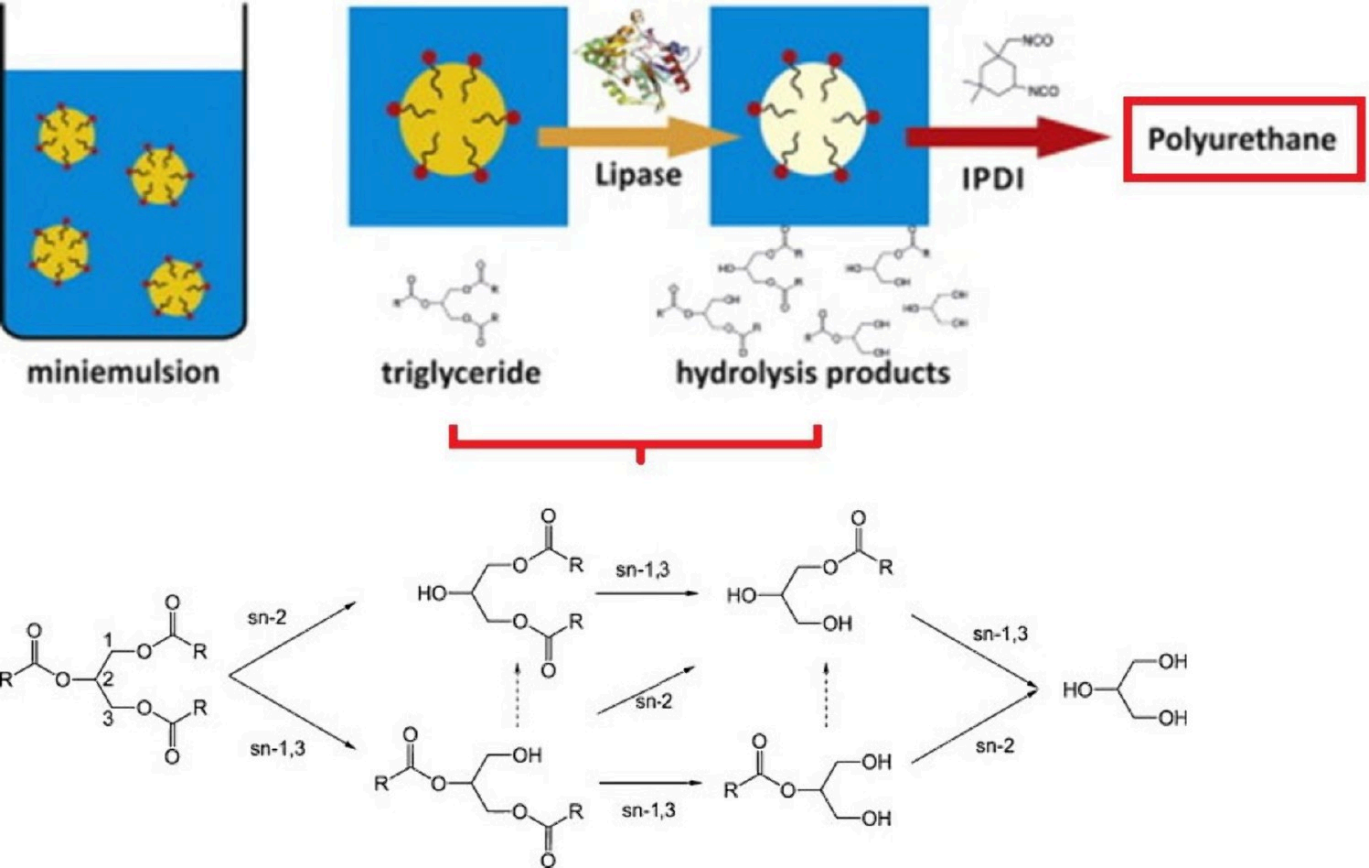
Hydrolyzed tricapyrlin (glycerol trioctanoate) used for polyurethane production. Recreated and reproduced with permission from ref (184). Copyright (2013), Elsevier.

Except for particular vegetable oils, most of the triacylglycerides do not include reactive functional groups which may contribute to the polyurethane structure synthesis. However, since all vegetable oil structures are esters, the appropriate functionalization can ensure their role in this material segment.^175^ The undesired hydrolysis (inevitable structural changes happening during the original application purposes) or the purposefully performed hydrolysis (to change the hydrophobic and unreactive structure of triacylglycerides) ensure vegetable oil structures’ reactive potential for polyaddition.^176,177^ The example of purposefully hydrolyzed vegetable oil’s analogue (tricapyrlin) used in the polyurethane industry is illustrated in Figure 8. The partially hydrolyzed glyceride possesses vacant −OH groups involved in the glycerol structure. These reactive centers can participate in the polyurethane-forming reaction, increasing their renewable-based content while ensuring the desired material properties.^175−177^ Also, the usage of secondary-produced materials such as waste cooking^178^ or cosmetic oil^38^ increases the sustainability production character by the valorization of the unwanted substances.

Several possibilities leading to the valorization of secondary products can be observed in the polyurethane-involving industry. Paruzel et al.^38^ incorporated a waste material into the polyurethane material by vegetable oil addition. Initially, solid-phase highly saturated coconut oil was used in the hydrolyzed form to be incorporated into the polyurethane systems based on the end-of-life valorization approach. Together with the car waste polycarbonate valorization, the partially hydrolyzed waste coconut oil served as a solvolysis reagent for polyurethane decomposition. Paciorek-Sadowska et al.^179^ experimentally produced polyurethane with different waste components. The secondary byproduct utilization was performed with the rapeseed oil manufacturing waste—the rapeseed cake. This industrial waste is obtained during rapeseed oil’s production. The solid-phase waste gathered during the rapeseed pressing can be incorporated into polyurethane-polyisocyanurate. Besides the incorporation of residual oil glycerides, this byproduct valorization targeted the biomass filler addition into the produced thermosets.

The purposeful oil hydrolysis leading to the substance participation in polyurethanes requires an additional step modifying the used triacylglyceride; however, the eventual properties can be regulated more precisely due to this approach compared to the simple waste valorization. The hydroxyl value is one of the most essential parameters for polyurethane manufacturing. The hydroxyl:isocyanate ratio is the key reactive parameter for polyaddition, since it determinates the eventual properties of the formed polymer.^180,181^ When −OH groups are present in the excess, the produced system tends to exhibit higher hydrophilic character due to the unbonded polar functional groups.^183^ Also, polyurethanes generated with a higher molar polyol ratio usually possess more flexibility.^182,183^ Contrary to these systems, rigid and semirigid polyurethanes include bigger isocyanate content.^182^ This approach produces the isocyanurate structure, ensuring the rigidity and toughness. Considering the functional group regulation, the directly tailored and produced glycerides with defined hydroxyl groups content can fulfill a wider application requirement compared to the simple waste valorization.

Next to the hydrolyzed and waste glycerides from various industrial sources, the crude obtained glycerol gained by the glycerides’ quantitative decomposition is often investigated and experimentally studied in the literature.^185−187^ Vegetable oils’ hydrolysis has a key significance for fuels and biodiesel production.^188^ The methyl esters of fatty acids are used for these purposes, while glycerol remains for additional purification and further manufacturing or can be directly incorporated to polyurethanes avoiding the purification. Since glycerol contains three hydroxyl groups directly next to each other, this substance primarily serves the production of the rigid and highly cross-linked polyurethane foams used in the construction industry (for example, ref (189)).

### 3.3 Transesterified and Functionalized Oils

**Figure 9:**
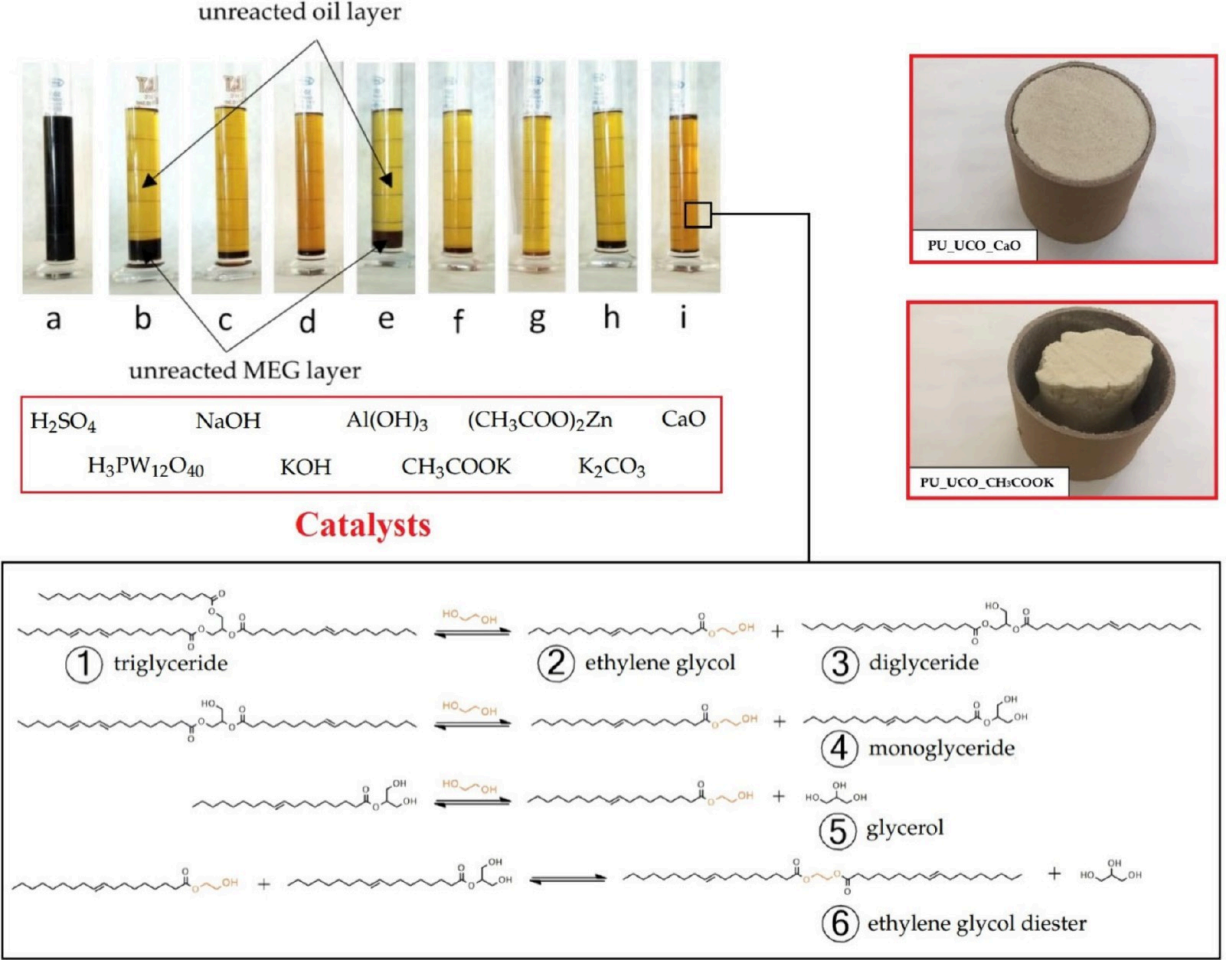
Transesterified cooking oil (incorporating different catalysts) used for the polyurethane formulation. Recreated and reprinted with permission under a Creative Commons (http://creativecommons.org/licenses/by/4.0/) from ref (203). Copyright (2022), MDPI.

The transesterification helps enormously with the functional determination of the final product’s properties. While pure castor oil or a simple hydrolyzed glyceride structure provides only a specific range of potential outcomes, rather than flexible castor oil or rigid polyurethanes using glycerides, the chemically modified oil structures enlarge the utility possibilities. Several polar structures may be suggested and produced using polyols with a high hydroxyl functional group number such as pentaerythritol.^190,191^ Such transesterified oil-based structures can serve as adhesives or surface active substances.^192^ The simultaneous hydrophobic character of the present fatty acid structure and the high polarity of numerous hydroxyl groups present in the compound ensures the unique application potential.^192^ On the other hand, fatty acid glycerides can be transesterified using double-functional alcohols such as ethylene glycol, diethylene glycol, or propylene glycol.^193−195^ Ethylene glycol application for the triacylglyceride transesterification study using different catalysts is shown in Figure 9. The hydroxyl functional group decrease leads to the flexibility enhancement, less cross-linking density, and increased chain extending properties.^183^

Several nitrogen-containing vegetable oil derivatives were described and studied in the literature.^196−198^ The ester bonding of the triacylglycerides may be transesterified using the polyol structure, the combination of amine and hydroxyl functional groups may also serve the functionalization, or aminolysis of the ester bonding may be performed to obtain modified fatty acid structures.^196−198^ Particular triethanolamine esters of vegetable oils or their diethanolamides were synthesized and studied for the polyurethane-producing purposes.^200^ Also, the combination of triacylglyceride’s aminolysis with the epoxidized fatty acid modification was performed to influence the eventual polyurethane properties from many standpoints.^199^ The present nitrogen atoms provided by particular functional groups within the polyurethane-forming reactants increases the material toughness due to the additional hydrogen bonding propagation and the inconsistent polar-involving structural character.^201,202^

### 3.4 Chemical Recycling Using Oils

The polyurethane glycolysis process was numerously described and studied in the available literature.^185−187^ The reaction of polyols with the formed urethane bonding, leading to the depolymeration of formed polyurethanes with the simultaneous production of unbonded alcohol released from the structure, has been used to produce raw materials for polyurethane chemical recycling approach.^204^ The reactive polyol is added into the polyurethane material and the polymer liquefaction occurs in the presence of an appropriate catalyst.^204^ The produced liquid raw material contains many unbonded hydroxyl groups and also particular content of the amine functional groups as the urethane bonding glycolysis occurs.^205^ The formed amine groups after the catalyzed depolymeration are often removed from the structure via deamination or the quantitative reaction with appropriate nucleophile such as anhydrides.^206,207^ Once the depolymerized structure is modified by deamination, the chemical recycling approach can be suggested since this system behaves similarly to the starting polyol from the reactivity standpoint.^206,207^

**Figure 10:**
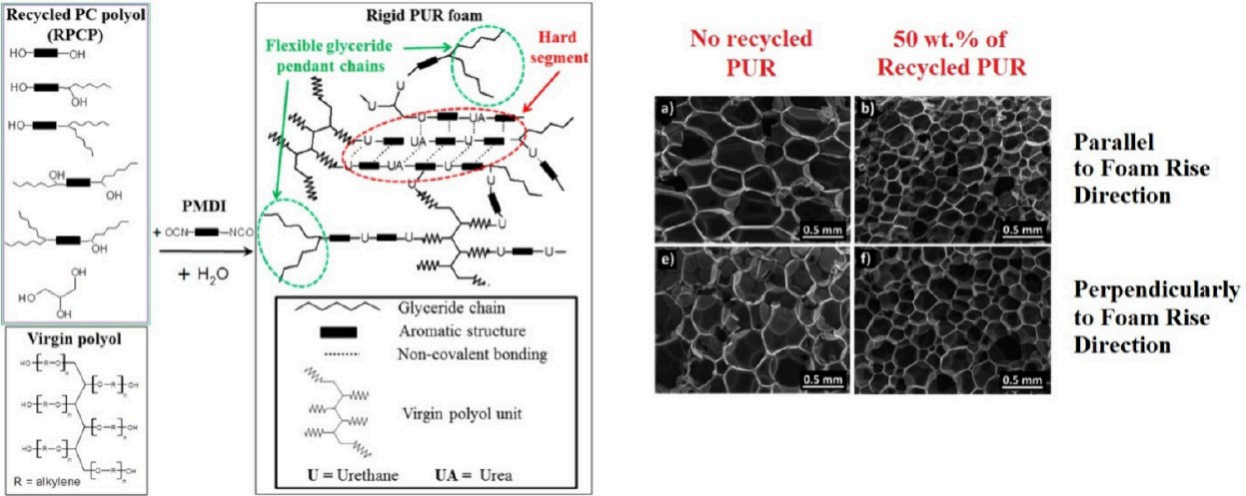
Rigid polyurethane foam fabricated from the coconut-oil-based glycerides and used polyurethane from vehicles chemically recycled via solvolysis. Reprinted with permission from ref (38). Copyright (2017), American Chemical Society.

The modified oils used for the potential chemical recycling of waste polyurethanes may ensure different eventual products’ properties. The high rigidity and toughness, resulting after small polyols incorporation such as glycerol or pentaerythritol,^208,209^ can be substituted for flexible character of chemically recycled polymers comprising of the reacted functionalized triacylglycerides.^210^ The particular approach verified on the vehicle end-of-life valorization was performed with coconut oil glycerides used toward the solvolysis or commercially used polyurethanes (see Figure 10).^38^ Also, the combination of castor oil’s backbone structure and the transesterification of its ricinoleic acids led to the industrially verified chemical recycling of vehicle car headliners used as a sound barrier in car ceiling. This process resulted in the produced polyurethanes’ flexibility and durability enhancement caused by the unique molecular structure of the transesterified castor oil using propylene glycol as the polyol for the functional substitution.^210^

## 4 Coatings

Vegetable oils have served coating purposes for decades. The naturally cross-linked unsaturated triacylglycerides applied to different substrates’ surfaces, mainly wood or paper,^211,212^ used the surrounding atmosphere oxygen to form a reactoplastic molecular structure, ensuring the mechanical protection or exterior decorations.^213,214^ Nowadays, several chemical functionalization approaches are suggested and realized to speed up the oil-involving coating process and avoid the slow oxidizing caused by the surrounding air. The applicability efficiency is ensured by the previously discussed chemical modifications: epoxidization, acrylation, methacrylation, or nucleophilic substitutions.^215−217^ Many of such functionalized triacylglyceride-containing systems can be polymerized via free radical or cationic polymerization^218^ activated by photoinitialization.^219^ These oil derivatives provide the fast product fabrication while ensuring a high sustainable character due to the majority of carbon from the renewable sources located in vegetable oil’s structure.^218,219^ These functionalized systems fulfill many different coating-providing objectives. The mechanical protection provided by rigid and though oil layers,^220^ barrier character preventing substrates’ soaking or wetting,^221^ anticorrosion treatment,^222^ or the ultraviolet irradiation protection^223^ are among the particular triacylglycerides utilities.

### 4.1 The Mechanical Protection

**Figure 11:**
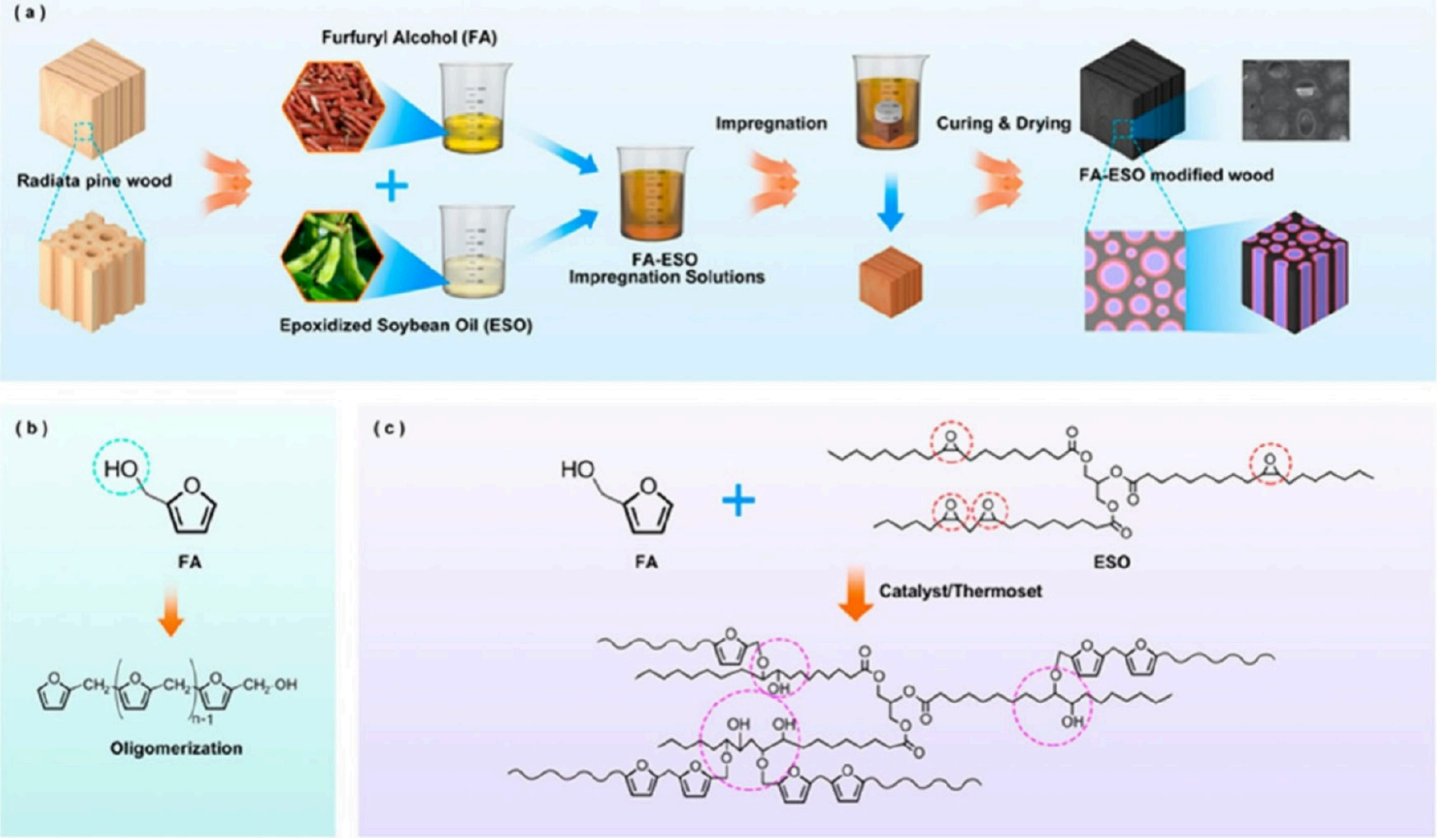
Whole impregnation of radiata pine wood scheme to obtain tough modified product. (a) Steps in FA–ESO (furfuryl alcohol–epoxidized soybean oil)-treated wood. (b) Oligomerization reactions of FA. (c) Proposed possible cross-linking reactions between FA and ESO. Reprinted with permission from ref (233). Copyright (2021), American Chemical Society.

The optimal adhesion, enforcing mechanical properties, and reasonable availability are the primary requirements for the protective coatings.^224^ Various substrates demand effective protection against stretching or other unoccasional damage.^225^ Typically, the wood substrates are ideal candidates for oil-based reactive coatings, and historically, the air-oxidized triacylglyceride layers were applied mainly on these materials.^226,227^ The wood chemical structure contains both polar hydroxyls occurring in cellulose and hemicelluloses, while lignin aromatic-containing content exhibits more nonpolar character.^228^ Vegetable oil’s backbone structure is enormously hydrophobic; however, since the functionalization such as epoxidization or the nucleophilic substitution comes into play, the triacylgyceride’s surface energy character changes.^229,230,235^ Both epoxy and hydroxyl functional groups increase the permanent dipole moment of oils’ carbon backbone, and the formed derivatives form molecular interactions with the substrate’s surface causing the enhanced adhesion and optimal layer-forming behavior.^231^ As a result, the additional vegetable oils’ functionalization increases the applicability and layer-forming process while also enhancing the adhesion toward certain substrates typically treated with these coatings.^232^ The vegetable oil-based wood impregnation for protective purposes is schematically and structurally illustrated in Figure 11. Wang et al.^301^ studied hydroxyl functional groups’ modification potential within the modified soybean oil for mechanical force-sensitive fields such as damage detection, antifalsification, or decorative purposes. The investigated system involved previously epoxidized and methoxylated triacylglycerides produced with vacant hydroxyl groups in the carbon backbone. The hydroxyl groups formed a polyurethane thermoset with incorporated spiropyran due to a polyaddition reaction with MDI.

### 4.2 Barrier Properties

The signature hydrophobic character of nonpolar vegetable oil’s carbon backbone provided by the occurring fatty acids in the structure promises the potential for the optimal water vapor barriers and antiwetting paper coatings.^234^ Previously discussed wood substrates also exhibit exceptional retention to water due to their molecular structure; however, paper materials fabricated from cellulose lack the lignin structures, and generally, paper sheets lose their mechanical properties enormously while exhibited to water compared to wood.^236^ Also, the food packaging fabricated from sustainable sources such as nanofibrillated cellulose (NFC) can take an advantage of functionalized triacylglycerides fabricated also from renewable sources and enhancing the hydrophobic properties of the eventual protective film.^237^ Since paper or thin-layer packaging materials involve a complication during the coating process due to the position instability caused by their low mass weight in combination with strong coating precursor adhesion during the layer forming, the viscosity modifiers (known as reactive diluents) can improve the fabrication process.^235^ The decreased viscosity helps with the reactive compound’s distribution across the targeted substrate and has the potential to modify the adhesion or enhance the systems reactivity to improve the coating process efficiency.^238^

### 4.3 Anticorrosion Effect

**Figure 12:**
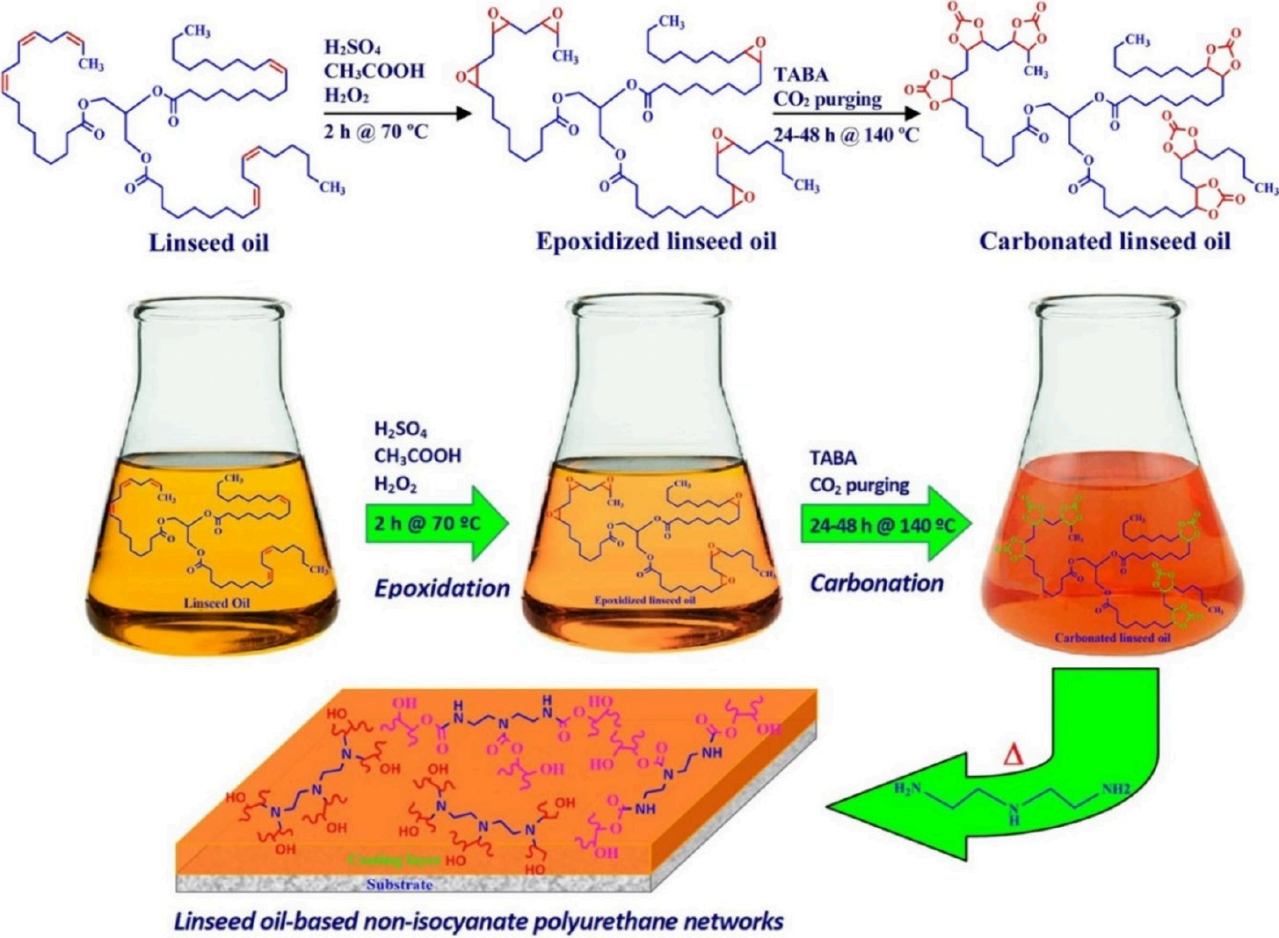
Nonisocyanate polyurethane oil-based coating for anticorrosion properties. Reproduced with permission from ref (246). Copyright (2021), Elsevier.

The corrosion turns pure metal materials into thermodynamically more stable metal compounds such as oxides, leading to the metal-fabricated products’ degradation and the properties’ profile changing.^239,240^ This process occurs spontaneously, while the particular object is exposed mainly by the water involving systems.^239^ Also, the electrochemical reactions increase the stable oxide formation.^241^ Due to the exceptional water-repelling properties that vegetable oils exhibit, the curable triacylglycerides make them ideal representatives to prevent this material-degrading process.^242,243^ The self-healing effect is strongly advantageous when the protective coatings against corrosion are considered.^244^ Since the adverse metal oxidizing can initiate in any layer defect, the surface regeneration is essential.^244^ The combination of different modified oils may ensure the self-healing effect to enhance the durability of the anticorrosion effect. Oktay et al.^245^ studied such a combined system for anticorrosion coatings. The cyclic anhydride (maleic anhydride)-modified vegetable oil in combination with epoxidized triacylglycerides can compose such a self-healing layer-forming system. The present unopened maleic anhydride cycles react with epoxy functional groups within the oil’s carbon backbone (dynamically at the elevated temperatures). This nucleophile substitution between two differently modified vegetable oils leads to the cross-linked molecular structure forming the anticorrosion protection. The temperature increase promotes layer regeneration when the mechanical damage affects the coated material. Generally, the anticorrosion coatings are studied via potentiodynamic polarization. Different electrodes and electrolytes are used for the electrochemical investigation, and the functional anticorrosion effect is exhibited when the applied current decreases with the rising potential on cathode or anode. This method is widely used to obtain corrosion effects in a shorter time compared to the spontaneous reaction in water solutions. The anticorrosion vegetable oil-based coating example using a nonisocyanate PUR thermoset is displayed in reaction and functional schemes in Figure 12.

### 4.4 Ultraviolet Protection

**Figure 13:**
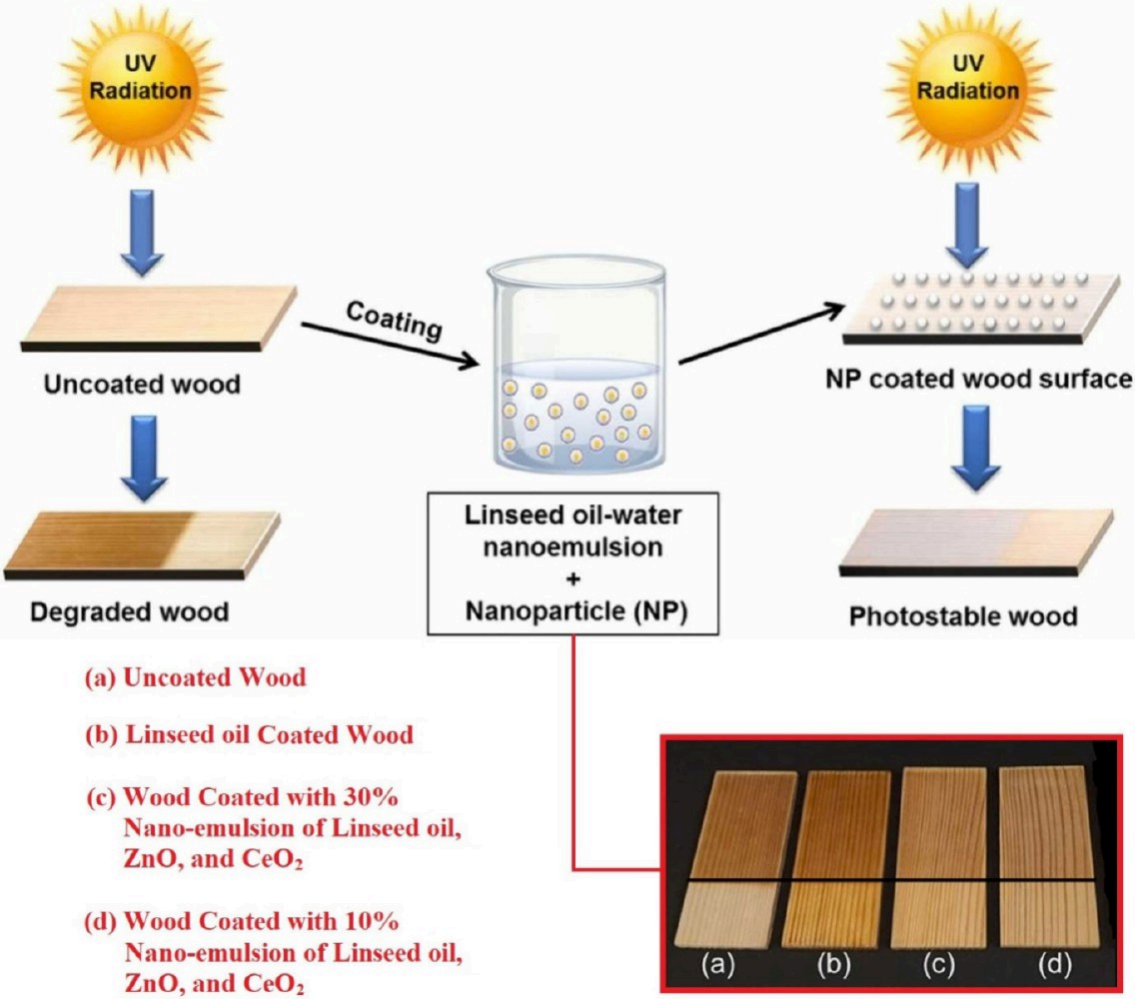
Linseed oil-coated wood substrates using zinc oxide (ZnO) and cerium oxide (CeO_2_) for UV-stabilization. Recreated and reproduced with permission from ref (255). Copyright (2022), Elsevier.

The ultraviolet irradiation causes several photodegradation processes, affecting many materials including plastic, colors, or wood products.^247−249^ The lumber-fabricated materials suffer considerable UV-initiated damage mainly due to the lignin presence in their complex molecular structure.^250^ The wood appearance changes caused by UV irradiation are shown in Figure 13. Next to cellulose and hemicelluloses, lignin enhances the toughness and strength of wood-containing products.^251^ However, the heterogeneous molecular structure of this component comprises many aromatic cycles containing several chromophores, which interact with the electromagnetic irradiation and absorb the energy leading to the electronic excitations.^252^ This process can be connected to many following processes such as the free radical formation or the generation of the de-excitation heat.^253^ Overall, the irradiation absorption leads to the structural changes within lumber-fabricated materials.^250^ The appropriate surface modifications can prevent fast UV-initiated degradation, since the irradiation affects the wood object through its surface. The modified transparent triacylglycerides containing epoxy or other reactive functional groups can fulfill the protective purpose.^254,255^ The oil’s structure is absent of any significant UV-sensitive chromophores, since it consists of the aliphatic hydrocarbon backbone possessing just ester or additional generated functional groups. The transparency provides the natural appearance of the coated wood products, while the UV-impenetrability protects the substrate from the degradation process.^254,255^ Currently, petroleum-based epoxides, such as diglycidyl ether of bisphenol A (DGEBA)^256^ or other glycidyl ethers,^257^ are widely used for the UV-protection purposes. Next to the sustainability enhancement, modified triacylglycerides possess adverse properties such as increased flammability or high material consumption caused by the enormous wood oil soaking.^258^ These circumstances can be overcome by appropriate additives preventing the flammability (the metal hydroxide-based, nitrogen-based, halogen-based intumescent-charring agents, or nanoparticle fire retardants)^259,260^ or decreasing the oil soaking (pretreatment wood coating).^261^

## 5 Adhesives

Oil-derived materials produced for attaching different substrates possess various carbon backbones and are comprised of numerous functional groups. A wide range of used adhesives are petroleum-based systems.^262,263^ The epoxy or acrylate derivatives are the most known and applied substances.^264−266^ As previously discussed, both mentioned chemical modifications can be performed with triacylglycerides. The reacting adhesives such as polymerizable acrylates and methacrylates form solid and cured layer to ensure the attaching purpose.^267^ The contact adhesion succeeds when noncovalent molecular interactions are formed between the adhesion-providing film and the substrate.^268^ The polyurethane-forming oil structures represent an enormous group of adhesion-providing compounds.^269−271^ Vegetable oil-based adhesives have two major advantages compared to the commercially used fossil-based products: the ecological footprint reduction and the beneficial economic factors.

### 5.1 Polyaddition Approach for Adhesives

As was discussed, polyurethanes require two key functional groups to form polymeric structures via polyaddition: the hydroxyl and isocyanate group.^269−271^ The modified vegetable oils are commonly investigated and experimentally studied regarding the petroleum-based-involving isocyanates for polyurethane syntheses.^278−284^ The triacylgricerides substitute the polyol role in the forming adhesive.^272^ The approaches leading to the hydroxyl group formation were described: the hydroxylation of epoxidized oils in acidic conditions forming the multifunctionalized vegetable oils,^273^ the general nucleophile substitution generating the secondary formed hydroxyl within the epoxidized structure,^274,275^ or the pure castor oil possessing the vacant hydroxyl can be incorporated into polyurethane systems.^276^ The specifically prepared vegetable oils serve as typical polyol raw materials for the eventual adhesives; additionally, the reactive functional groups in the carbon backbone structure, such as acrylates or methacrylate, can contribute to the eventual attaching purposes via photoinitial process (for example, see ref (277)).

**Figure 14:**
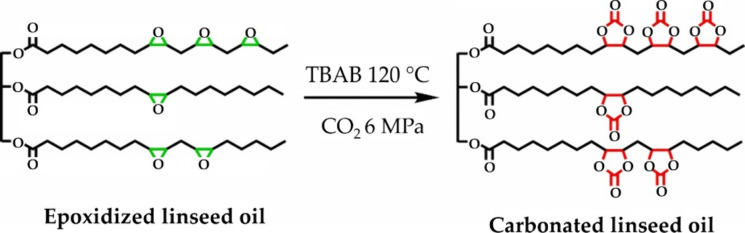
Carbonated linseed oil synthesis for the nonisocyanate polyurethane adhesive production. Recreated and reprinted with permission under a Creative Commons (http://creativecommons.org/licenses/by/4.0/) from ref (300). Copyright (2024), MDPI.

The typical TDI or MDI can represent the isocyanate-containing structure mandatory for polyurethane-generating adhesives. Numerous modified vegetable oil systems reacting with conventional isocyanates were reported and are published in the literature (Table 4). The nonisocyanate polyurethanes attract a vast amount of attention since the functionalized triacylglycerides can be produced entirely from renewable sources. The conventional systems such as TDI or MDI require phosgene for their synthesis.^285,286^ On the other hand, the carbonated structures produced from epoxidized vegetable oils involving high temperatures and CO_2_ levels connected to the high-pressure procedure (as schematically summarized in Figure 14) can represent a substitute to the conventionally used fossil-based and toxic reactants.^287,300^ Such functionalized structures react with amines, leading to the solid-forming products used in the adhesive industry. The various molecular tailorings of the eventual polyurethane using nonisocyanate templates is the main benefit of such systems. The remaining and generated functional groups in the compounds’ structures provide the desired water-resistant or hydrophilic character of the adhesive determining the eventual application.^287,300^

**Table 4:** Different Commercial Isocyanate-Involving Polyurethanes Using Modified Vegetable Oils as Polyols

Oil type	Isocyanate type	NCO:OH ratio	Application	Reference
Castor oil (transesterified by glycerol)	MDI	1.0–1.4	TiO_2_ filler effect study	(278)
Palm oil polyester	pMDI, TDI	1.3, 1.5	Wood adhesive	(279)
Castor oil	MDI	1.0–3.0	Wood adhesive	(280)
Epoxidized soybean oil	pMDI	1.5	Wood adhesive	(284)
Castor oil	HMDI	1.87, 3.20	Wood adhesive	(281)
Soybean oil polyol	IPDI	1.02	Electronic, automotive	(282)
Castor oil	PPI	1.15	Conductive adhesive	(283)

### 5.2 Radically Initiated Adhesives

Next to many other applications mentioned previously, the acrylate and methacrylate vegetable oil derivatives can be turned into adhesive materials.^288,289^ The chemistry of petroleum-based and biobased acrylate and methacrylate glues and attaching materials is identical: the free radical polymerization occurs once the appropriate initiator gets in contact with the acrylate of methacrylate functional groups.^290^ The aerosol-forming peroxides serve as the hardeners for these adhesives.^291^ Numerous curable systems follow this chemical process. The pressure-sensitive adhesives (PSA) are a different group of compounds. These systems do not include liquid systems changing their physical state to cured solid layers. PSAs typically contain sticky working segments composed of the modified substrate’s surface or nonvolatile oligomer compounds possessing the ideal properties for the contact adhesion.^288,292−295^ These adhesives do not involve any chemical reaction such as curing of polyaddition (polyurethane adhesives), and the simple applied pressure and optical surface contact provide the attaching effects.^293^ The directly synthesized acrylated/methacrylate vegetable oils or the systems produced via emulsion polymerization and then applied as adhesives are investigated and studied in the literature.^296−298^

### 5.3 Nucleophilic Substitution and Ring-Opening Approach for Adhesives

**Figure 15:**
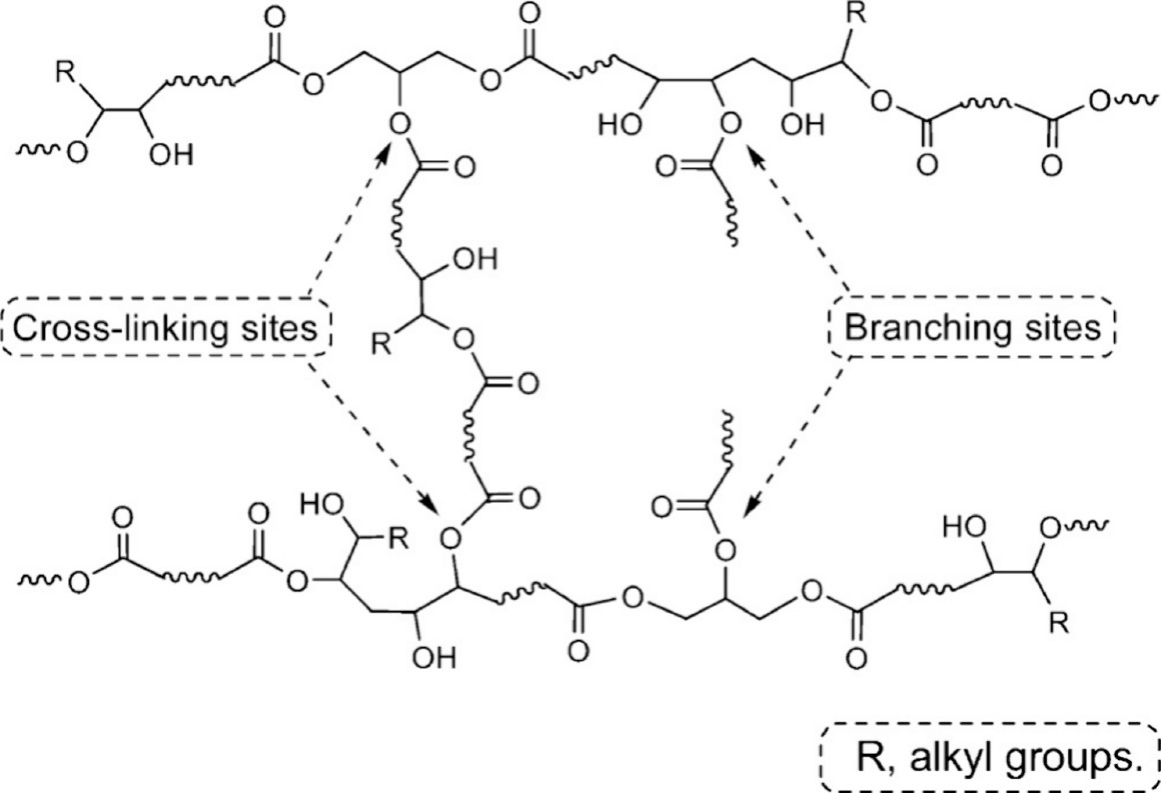
Schematic chemical structure of epoxidized oil based adhesive cured with carboxylic diacid. Reprinted with permission from ref (299). Copyright (2014), American Chemical Society.

The multihydroxylated hydrophobic structures are typically used as entering materials for hydroxylation,^70^ ring-opening polymerization-based materials,^54^ nucleophile substitution,^34^ or a cationic polymerization precursor.^34^ In the field of adhesives, the two component reactive systems were investigated to serve as PSA. Li and Li^299^ published work regarding the epoxidized vegetable curing with carboxylic diacids (see the chemical structure in Figure 15). The epoxidized soybean oil was prepolymerized using difunctional carboxylic acids (dimer hydrogenated acid, adipic acid, and sebatic acid) mixed at elevated temperature (85 °C) to produce a highly viscous system to be coated on the supportive substrate. Then, the PSA preparation followed involving the preoligomer distribution onto the paper sheet and the curing process at 160 °C. The standard nucleophile substitution occurred at elevated temperatures without any catalyst. This process using reactive epoxy functional groups was reported previously. Eventually, the PSA was produced entirely from the renewable materials as adipic/sebatic acids, and the vegetable epoxidized oil can be synthesized from biobased sources.

## 6 Conclusion

Vegetable oils exhibit specific hydrophobic character due to their unique molecular structure composed of fatty acids absent of polar functional groups such as hydroxyl, amines, or unbonded carboxyl. Most of the vegetable triacylglycerides contain unsaturated double bonds, determining their physical–chemical properties and ensuring a material-creating potential. The double bonds can be modified to several functional groups such as epoxy, hydroxyl, or carbonate, promising a wide variety of future applications. Particular vegetable oils (castor oil) naturally comprise the reactive groups in their structure naturally. The functionalization was performed and led to numerous utilities in the material chemistry field such as additive manufacture, the polyurethane industry, coatings, and adhesives. The main vegetable oils’ application potential lies in the availability and sustainability of such substances. Several secondary glycerides from waste food or cosmetic industries were obtained and incorporated into the added-value products, which ensures a sustainable approach linked to the currently performed processes. This approach evaluates the disposed materials and uses the advantages of the oil’s molecular structure. The continual application of wastes and secondary products not only solves the issues connected to the environmental safety (waste reduction), but also the material-producing strategies reduce the expenses invested into entering materials which are substituted. Other utilities using primary produced triacylglycerides can benefit from this entering reactant’s availability across the globe. Generally, the oil-containing materials reduce the ecological footprint due to the application of materials from renewable sources. While different sources of vegetable oils are used, this Review proves the reported and verified utility of particular triacylglycerides obtainable in the specific agricultural areas.

Triacylglycerides represent a complex group of variously structurally defined long carbon chain compounds with hydrophobic character and high renewable carbon content. These properties promise an extensive potential in many kinds of material manufacture, since the petroleum-based compound utilities tend to be limited by legislature. The native ester form of vegetable oils is beneficial for additive manufacture, highly flexible polyurethanes, or oil-incorporating protective coatings. On the other hand, the promising outlook for hydrolyzed, functionally selected, and degraded glycerides lies in the chemical recycling of polyurethanes or their utility in emulsion–polymerization processes. Generally, fatty-acid-containing materials primarily exhibit flexible character, high thermal stability, and exceptional hydrophobicity. These properties ensure a potential vegetable oil compound substitution in highly viscous systems in additive manufacture, the flexibility-required polyurethanes, biobased substrates for coatings, or the primarily hydrophobic adhesives.
